# Supplementary material for: Radiotherapy for Clinically Localized T3b or T4 Very-High-Risk Prostate Cancer-Role of Dose Escalation Using High-Dose-Rate Brachytherapy Boost or High Dose Intensity Modulated Radiotherapy
Source: Cancers (Basel). 2021 Apr 13;13(8):1856. doi: 10.3390/cancers13081856 (PMC8070084; doi:10.3390/cancers13081856)
Supplement: Supplementary file 1 [file cancers-13-01856-s001.zip › cancers-1140449-supplementary.pdf]

Supplementary Materials:

Radiotherapy for Clinically Localized T3b or T4 Very-High-Risk Prostate Cancer-Role of Dose Escalation Using High-Dose-Rate Brachytherapy Boost or High Dose Intensity Modulated Radiotherapy

Hideya Yamazaki, Gen Suzuki, Koji Masui, Norihiro Aibe, Daisuke Shimizu, Takuya Kimoto, Daisuke Shimizu, Ken Yoshida, Satoaki Nakamura and Haruumi Okabe

Table S1. Patients characteristicsamong three groups.

**Variables**	**Strata**	**Conv. EBRT (*n* = 86)**		**High dose EBRT (*n* = 39)**		**HDR boost (*n* = 124)**		***p* - value**
		**No. or Median (range)**	(%)	No. or Median (range)	(%)	No. or Median (range)	(%)	
Age		70.5 (60–89)		73 (64–79)		71 (60–86)		0.2262
T category	3b	67	-78%	34	-87%	115	-93%	
	4	19	-22%	5	-13%	9	-7%	0.0077
iPSA	(ng/mL)	42.07 (3.4–398)		27.2 (4.7–240)		31.9（4.73–486）		0.3398
Gleason score	-6	0	0%	4	-10%	9	-7%	
	7	34	-40%	11	-28%	53	-43%	
	8-	51	-59%	24	-62%	62	-50%	**0.0383**
Prescribed dose (EQD 2Gy)	(Gy)	72 (70–72)		78 (74–80)		108.7 (104.1–129.8)		**<0.0001**
Hormonal therapy	Yes	86	-100%	38	-97%	121	-98%	0.3415
Neoadjuvant	months	7.5 (2–18)		6.5 (5–14)		12 (1–74)		**<0.0001**
Adjuvant	months	20 (1–30)		23 (3–60)		36 (8–114)		**<0.0001**
	No	0	0%	1	-3%	3	-2%	
Follow-up	(Months)	77.5 (18.7–135)		62.76 (24–87)		64 (13–153)		0.0002

Table S2. Detailed BED and EQD2Gy for each treatment schedule.

Prescribed dose	PT No.	(%)	BED(α/β=1.5Gy)	(EQD 2Gy)
Conv. EBRT (n=86)				
70Gy/35fr	14	(16%)	163	70
72Gy/36fr	72	(84%)	168	72
High dose EBRT (n=39)				
74 Gy/36fr	12	(31%)	172	74
78Gy/39fr	14	(36%)	182	78
74.8Gy/34fr	8	(21%)	184	79
80Gy/40fr	4	(10%)	186	80
70Gy/28fr	1	(3%)	186	80
HDR boost (n = 124)				
18 Gy / 2 fr +EBRT 39 Gy /13 fr	10	(8%)	243	104
18 Gy / 2 fr +51 Gy / 17fr	14	(11%)	279	119
18 Gy / 2 fr + 48 Gy/16fr	1	(1%)	270	115
20 Gy / 2fr + EBRT46Gy /23fr	4	(3%)	260	111
21 Gy /2 fr + EBRT 45 Gy /15fr	4	(3%)	303	130
21Gy / 3fr+ EBRT 51 Gy / 17 fr	1	(1%)	272	116
31.5 Gy / 5fr + EBRT 30 Gy /10fr	90	(73%)	253	109

BED=n × d × (1 + d/(α/β)) ; (α/β=1.5 Gy, *n*=fraction number, d = single dose).

EQD 2Gy=n × d × (α/β + d)/(α/β + 2).

Table S3. Acute toxicity among three groups.

**Toxicities**	**Grade**	**Conv EBRT. (*n* = 86)**		**HDEBRT (*n* = 39)**		**HDR boost (*n* = 124)**		***p*-value**
		**No.**	(%)	No.	(%)	No.	(%)	
**Genitourinary**	0	33	-38%	26	-67%	36	-29%	**<0.0001**
	1	48	-56%	9	-23%	66	-53%	
	2	5	-6%	4	-10%	22	-18%	
	3	0	0%	0	0%	0	0%	
**Gastrointestinal**	0	45	-52%	37	-95%	113	-91%	**<0.0001**
	1	40	-47%	2	-5%	11	-9%	
	2	1	-1%	0	0%	0	0%	
	3	0	0%	0	0%	0	0%	

Table S4. Late toxicity among three groups.

**Toxicities**	**Grade**	**Conv EBRT. (*n* = 86)**		**HDEBRT (*n* = 39)**		**HDR boost (*n* = 124)**		***p*-value**
		**No.**	(%)	No.	(%)	No.	(%)	
**Genitourinary**	0	69	-80%	36	-92%	56	-45%	**<0.0001**
	1	8	-9%	1	-3%	54	-44%	
	2	5	-6%	2	-5%	7	-6%	
	3	4	-5%	0	0%	7	-6%	
**Gastrointestinal**	0	65	-76%	35	-90%	110	-89%	0.0741
	1	18	-21%	2	-5%	12	-10%	
	2	1	-1%	2	-5%	1	-1%	
	3	1	-1%	0	0%	1	-1%	
